# Reporting guidelines for modelling studies

**DOI:** 10.1186/1471-2288-12-168

**Published:** 2012-11-07

**Authors:** Carol Bennett, Douglas G Manuel

**Affiliations:** 1 Clinical Epidemiology Program, Ottawa Hospital Research Institute, 1053 Carling Avenue, Ottawa, K1Y 4E9, Canada; 2 ICES@uOttawa, Institute for Clinical Evaluative Sciences, 1053 Carling Avenue, Ottawa, K1Y 4E9, Canada; 3 Department of Family Medicine, University of Ottawa, Ottawa, K1H 8M5, Canada; 4 CT Lamont Primary Health Care Research Centre, University of Ottawa, Ottawa, ON, Canada; 5 Bruyere Research Institute, University of Ottawa, Ottawa, ON, Canada; 6 Department of Epidemiology and Community Medicine, The University of Ottawa, Ottawa, ON, Canada

## Abstract

**Background:**

Modelling studies are used widely to help inform decisions about health care and policy and their use is increasing. However, in order for modelling to gain strength as a tool for health policy, it is critical that key model factors are transparent so that users of models can have a clear understanding of the model and its limitations.Reporting guidelines are evidence-based tools that specify minimum criteria for authors to report their research such that readers can both critically appraise and interpret study findings. This study was conducted to determine whether there is an unmet need for population modelling reporting guidelines.

**Methods:**

We conducted a review of the literature to identify: 1) guidance for reporting population modelling studies; and, 2) evidence on the quality of reporting of population modelling studies. Guidance for reporting was analysed using a thematic approach and the data was summarised as frequencies. Evidence on the quality of reporting was reviewed and summarized descriptively.

**Results:**

There were no guidelines that specifically addressed the reporting of population modelling studies. We identified a number of reporting guidelines for economic evaluation studies, some of which had sections that were relevant population modelling studies. Amongst seven relevant records, we identified 69 quality criteria that have distinct reporting characteristics. We identified two papers that addressed reporting practices of modelling studies. Overall, with the exception of describing the data used for calibration, there was little consistency in reporting.

**Conclusions:**

While numerous guidelines exist for developing and evaluating health technology assessment and economic evaluation models, which by extension could be applicable to population modelling studies, there is variation in their comprehensiveness and in the consistency of reporting these methods. Population modelling studies may be an area which would benefit from the development of a reporting guideline.

## Introduction

Modelling studies are used widely to help inform decisions about health care and policy and their use is increasing [1,2]. A model is “an analytical methodology that accounts for events over time and across populations, that is based on data drawn from primary or secondary sources…” and in the context of health care-evaluation “…whose purpose is to estimate the effects of an intervention on valued health consequences and costs” [3]. Its value lies not only in its results, but also in its ability to reveal the connections between its data and assumptions and model outputs [3]. But, as pointed out by Garrison, models don’t have to be mathematically sophisticated to be hard to follow [4]. For these reasons, a model should not be a “black box” for the end-user but be as transparent as possible [3].

To address the problem of poorly reported research, multiple reporting guidelines have been developed and validated for use with a number of study designs. Reporting guidelines are evidence-based tools that employ expert consensus to specify minimum criteria for authors to report their research such that readers can both critically appraise and interpret study findings [5,6]. The EQUATOR Network, an international initiative whose aim is to improve the reliability of medical research by promoting transparent and accurate reporting of research studies, indexes more than 100 reporting guidelines on their Web site (http://www.equator-network.org).

The growth in the number and range of reporting guidelines has prompted guidance on how to develop one using a well-structured development process [6]. This study addresses the needs assessment-that is, to determine whether there is a need for population modelling reporting guidelines. More specifically, the objectives of our study were: to locate and assess any existing reporting guidelines for population modelling studies; to identify key quality criteria for the reporting of population modelling studies; and to determine if and how these criteria are being reported in the literature.

## Methods

We began this process with a search of the MEDLINE electronic database (MEDLINE (1950 – February 2011) via Ovid. Our electronic search strategy (see appendix), developed in consultation with a library scientist, was pragmatically designed to avoid being overwhelmed with irrelevant records. We hand-searched the reference lists and used the *related articles* feature in PubMED for all papers meeting our eligibility criteria. In addition, we reviewed relevant textbooks and Web sites. One reviewer screened the titles and abstracts of all unique citations to identify papers that met our inclusion criteria—that is, English language papers that provided explicit guidance on the reporting of population modelling studies or provided evidence on the quality of reporting of population modelling studies in the health science literature. The full-text report of each record passing title/abstract screening was retrieved and reviewed by the research team and its inclusion/exclusion status was established.

For records that provided explicit guidance on reporting of population modelling studies, the list of criteria identified was analysed using a thematic approach and the data was summarised as frequencies. For those papers that presented evidence on the quality of reporting of population modelling studies, we identified the aspects of reporting that were assessed and summarised the results descriptively.

## Results and discussion

**Figure 1:**
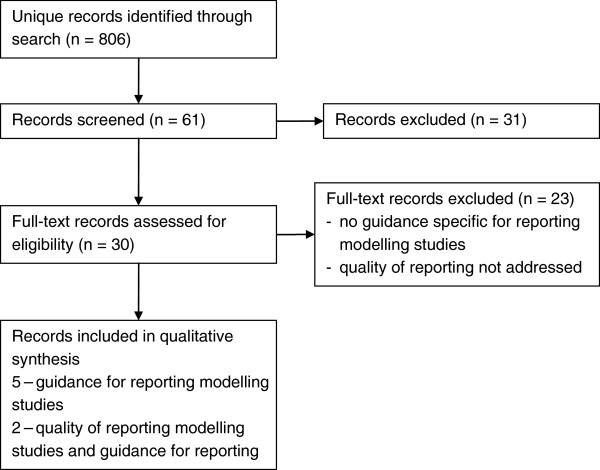
Flow diagram of records – guidelines for reporting modelling studies and evidence on the quality of reporting of modelling studies.

We identified 806 unique records through our search strategy, 30 full-text articles were reviewed to determine eligibility (Figure 1).

### Existence of guidelines for modelling studies

There were no guidelines that specifically addressed the reporting of population modelling studies. However, there were a number of reporting guidelines for economic evaluation studies: one of which was related to modelling [7] and one included a section which focused on the generalisability of modelling studies [8]. Additionally, we identified one paper that provided reporting guidance for a specific aspect of simulation modelling methodology – calibration [9].

Numerous guidelines have been published defining good practice for the conduct of economic evaluations in general and model-based evaluations in particular. We identified two papers that provided guidance for assessing the quality of decision-analytic modelling studies [3,10] and one paper that provided guidance for assessing validation of population-based disease simulation models [2].

### Identification of key reporting items

Amongst the relevant records that were analysed, we identified 69 quality criteria that have distinct reporting characteristics (Table 1).

**Table 1:** Checklist items for reporting modelling studies

**Dimension of Quality**	**Reporting item**	**Philips**	**Unal**	**ISPOR**	**Nuijten**	**Stout**	**Drummond**	**Kopec**
**STRUCTURE**								
Decision problem/objective	Is there a clear statement of the decision problem?	x			x		x	
	Is the objective of the evaluation specified and consistent with the stated decision problem?	x			x			
	Is the primary decision-maker specified?	x					x	
Scope/perspective	Is the perspective of the model clearly stated?	x		x	x			
	Are the model inputs consistent with the stated perspective?	x					x	
	Are definitions of the variables in the model justified?							x
	Has the scope of the model been stated and justified?	x						x
	Are the outcomes of the model consistent with the perspective, scope and overall objective of the model?	x						
Rationale for structure	Is the structure of the model consistent with a coherent theory of the health condition under evaluation?	x		x				x
	Are the sources of data used to develop the structure of the model specified?	x						
	Are the causal relationships described by the model structure justified appropriately?	x						
Structural assumptions	Are the structural assumptions clearly stated and justified?	x	x	x	x			
	Are the structural assumptions reasonable given the overall objective, perspective and scope of the model?	x						
Strategies/comparators	Is there a clear definition of the options under evaluation?	x		x	x			
	Have all feasible and practical options been evaluated?	x						
	Is there justification for the exclusion of feasible options?	x						
Model type	Is the chosen model type appropriate given the decision problem and specified causal relationships within the model?	x			x			x
Time horizon	Is the time horizon of the model sufficient to reflect all important differences between options?	x		x	x			
	Are the time horizon of the model, the duration of treatment and the duration of treatment effect described and justified?	x						
Disease states/pathways	Do the disease states (state transition model) or the pathways (decision tree model) reflect the underlying biological process of the disease in question and the impact of the interventions?	x		x	x			
Cycle length	Is the cycle length justified?	x		x	x			
Parsimony	Is there indication that the structure of the model is as simple as possible and that any simplifications are justified?			x				
**DATA**								
Data identification	Are the data identification methods transparent and appropriate given the objectives of the model?	x		x	x			
	Are results reported in a way that allows the assessment of the appropriateness of each parameter input and each assumption in the target settings?						x	
	Where choices have been made between data sources, are these justified appropriately?	x					x	x
	Where data from different sources are pooled, is this done in a way that the uncertainty relating to their precision and possible heterogeneity is adequately reflected?						x	
	Are the data used to populate the model relevant to the target audiences (i.e., decision-makers) and settings?						x	
	Has particular attention been paid to identifying data for the important parameters in the model?	x						
	Has the quality of the data been assessed appropriately?	x	x					x
	Where expert opinion has been used, are the methods described and justified?	x		x	x			x
Data modelling	Is the data modelling methodology based on justifiable statistical and epidemiological techniques?	x		x				
Baseline data	Is the choice of baseline data described and justified?	x						
	Are transition probabilities calculated appropriately?	x		x	x			
Treatment effects	If relative treatment effects have been derived from trial data, have they been synthesized using appropriate techniques?	x		x				
	Have the methods and assumptions used to extrapolate short-term results to final outcomes been documented and justified? Have alternative assumptions been explored through sensitivity analysis?	x		x	x			
	Have assumptions regarding the continuing effect of treatment once treatment is complete been documented and justified? Have alternative assumptions been explored through sensitivity analysis?	x						
Risk factors	Has evidence supporting the modeling of risk factors as having an additive or multiplicative effect on baseline probabilities or rates of disease incidence or mortality been presented?			x				
Data incorporation	Have all data incorporated into the model been described and referenced in sufficient detail?	x		x	x			
	Has the use of mutually inconsistent data been justified (i.e., are assumptions and choices appropriate)?	x						
	Is the process of data incorporation transparent?	x		x				
	If data have been incorporated as distributions, has the choice of distribution for each parameter been described and justified?	x		x				
	If data have been incorporated as distributions, is it clear that second order uncertainty is reflected?	x		x				
Assessment of uncertainty	Have the four principal types of uncertainty been addressed?	x						
	If not, has the omission of particular forms of uncertainty been justified?	x						
Methodological	Have methodological uncertainties been addressed by running alternative versions of the model with different methodological assumptions?	x						
Structural	Is there evidence that structural uncertainties have been addressed via sensitivity analysis?	x		x				
Heterogeneity	Has heterogeneity been dealt with by running the model separately for different subgroups?	x		x				
Parameter	Are the methods of assessment of parameter uncertainty appropriate?	x		x	x		x	x
	If data are incorporated as point estimates, are the ranges used for sensitivity analysis stated clearly and justified?	x	x					
	Which sensitivity analyses were carried out?		x					
**CONSISTENCY**								
Internal consistency	Is there evidence that the mathematical logic of the model has been tested thoroughly before use?	x		x				x
External consistency	Are any counterintuitive results from the model explained and justified?	x		x	x			
	If the model has been calibrated against independent data, have any differences been explained and justified?	x	x					
	How was the model calibrated?		x					
	Calibration - description of source data					x		x
	Calibration - description of search algorithm					x		x
	Calibration - description of goodness-of-fit metric					x		x
	Calibration - description of acceptance criteria					x		x
	Calibration - description of stopping rule					x		x
	Have the results of the model been compared with those of previous models and any differences in results explained?	x		x	x			x
**VALIDITY**								
Output plausibility	Has evidence of face validity - evaluation by experts in the subject matter area for a wide range of input conditions and output variables, over varying time horizons – been presented?							x
Predictive validity	Was the validity of the model tested?		x		x	x		x
	Is there a description of how the validity of the model was checked?		x			x		
	How was the validity quantified? (e.g., % explained)		x					
**COMPUTER IMPLEMENTATION**	Is the software used in the study listed and its choice justified?		x		x			x
**TRANSPARENCY**	Is the model available to the reader?		x					
	Is a detailed document describing the calibration methods available?					x		
	Do the authors provide relevant appendices?				x			
**SPONSORSHIP**	Is disclosure of relationship between study sponsor and performer of the study provided?				x			

We identified 22 items relating to the structure of the model and broadly classified them into 10 domains: 1) statement of decision problem/objective; 2) statement of scope/perspective; 3) rationale for structure; 4) structural assumptions; 5) strategies/comparators; 6) model type; 7) time horizon; 8) disease states/pathways; 9) cycle length; and, 10) parsimony.

We identified 28 items related to data issues and broadly classified them into 11 domains: 1) data identification; 2) data modelling; 3) baseline data; 4) treatment effects; 5) risk factors; 6) data incorporation; 7) assessment of uncertainty; 8) methodological; 9) structural; 10) heterogeneity; and, 11) parameter.

We identified 14 items related to consistency (internal and external) and validity (output plausibility and predictive validity). The final five items fell under computer implementation, transparency or funding.

The items are not mutually exclusive, and there is overlap if one takes into account implicit and explicit considerations. Even considering this, the records differed in terms of their comprehensiveness and the areas of model quality they considered. No item was identified by all of the resources, one item appeared in five lists, four items appeared in four lists, three items appeared in 17 lists and the remainder of the items appeared in only one or two lists (Table 1).

### Quality of reporting

We identified two papers that addressed reporting practices of modelling studies, the first of which was a systematic review of coronary heart disease policy models [11].

The authors evaluated 75 papers on the basis of whether a sensitivity analysis was carried out, the validity was checked, data quality was reported, illustrative examples were provided, if the model was potentially available to the reader (transparency), and if potential limitations were specified or discussed. This evaluation was based on authors reporting on the specific item in the articles.

Relatively few papers included in the review reported on quality issues: sensitivity analysis and assessment of validity were reported in very few models, 33% provided illustrative examples, working versions of the model were available in 10%, and 19% reported on limitations of their methodology,

The second paper examining the reporting practices of modelling studies looked more specifically at the reporting of calibration methods in 154 cancer simulation models [9]. Data elements abstracted included whether model validation was mentioned (52%) and if a description of the calibration protocol was provided (66%). The authors further characterized calibration protocols by five components. A description of the data used as calibration targets was reported by 95% of the studies and goodness-of-fit metrics were reported in 54% of the studies. However, the search algorithm used for selected alternative parameter values, the criteria for identifying parameter sets that provide an acceptable model fit, and the stopping criteria were not well reported (quantitative values not provided).

Few studies were identified that addressed the quality of reporting of population modelling studies. Overall, with the exception of describing the data used for calibration, there is little consistency in the reporting of items that have been identified as key quality items.

## Conclusions

Population modelling studies can fill an important role for policy makers. Their ability to synthesize data from multiple sources and estimate the effects of interventions can be invaluable, especially in areas where primary data collection may be infeasible. However, in order for modelling to gain strength as a tool for health policy, it is critical that key model factors are made transparent so that users of models have a clear understanding of the model and its limitations.

While numerous guidelines exist for developing and evaluating health technology assessment and economic evaluation models, which by extension can be applicable to population modelling studies, there is variation in their comprehensiveness and in the consistency of reporting these methods. There is evidence to suggest that key items are under-reported.

In other areas where reporting guidelines have been developed, there has been a favourable impact on the transparency and accuracy of reporting [12-15]. Population modelling studies may be another area which would benefit from the development of a reporting guideline. Moher and colleagues have outlined the importance of a structured approach to the development of reporting guidelines [6]. This paper provides results from initial steps in this structure approach. Future work should focus on identifying key information related to potential sources of bias in population modelling studies and identifying a multidisciplinary expert panel to steer the guideline development process.

## Appendix

### Search strategy

1. "Reproducibility of Results"

2. Quality control/

3. ((valid$ or reliab$ or quality or accura$) adj2 (result$ or report$ or data)).tw.

4. (good adj1 practice$).tw.

5. Guidelines as Topic/

6. (guideline$ or checklist$).tw.

7. or/1-6

8. (model$ adj3 (stud$ or method$ or process$ or simulation)).tw.

9. (modelling or modeling).tw.

10. 8 or 9

11. Research design/

12. Decision Support Techniques/

13. published literature.tw.

14. Research/

15. or/11-14

16. 16 7 and 10 and 15

## Competing interests

The authors declare that they have no competing interests.

## Authors’ contributions

Concept and design (CB, DM); acquisition of data (CB); analysis and interpretation of data (CB, DM); drafting of the manuscript (CB); critical revision of the manuscript for important intellectual content (CB, DM); and final approval of the version to be published (CB, DM). Both authors read and approved the final manuscript.

## Pre-publication history

The pre-publication history for this paper can be accessed here:

http://www.biomedcentral.com/1471-2288/12/168/prepub
